# Long-Term Systemic Treatment of a Mouse Model Displaying Chronic FSHD-like Pathology with Antisense Therapeutics That Inhibit *DUX4* Expression

**DOI:** 10.3390/biomedicines10071623

**Published:** 2022-07-07

**Authors:** Ngoc Lu-Nguyen, George Dickson, Alberto Malerba, Linda Popplewell

**Affiliations:** 1 Department of Biological Sciences, School of Life Sciences and the Environment, Royal Holloway University of London, Egham TW20 0EX, UK; ngoc.lu-nguyen@rhul.ac.uk (N.L.-N.); g.dickson@rhul.ac.uk (G.D.); 2 National Horizons Centre, Teesside University, Darlington DL1 1HG, UK

**Keywords:** FSHD, DUX4 inhibition, systemic antisense therapy, ACTA1-MCM/FLExDUX4

## Abstract

Silencing the expression of the double homeobox 4 (*DUX4*) gene offers great potential for the treatment of facioscapulohumeral muscular dystrophy (FSHD). Several research groups have recently reported promising results using systemic antisense therapy in a transgenic small animal model of FSHD, the ACTA1-MCM/FLExDUX4 mouse model. However, the treatment was applied in non-*DUX4*-induced mice or shortly after *DUX4* activation, which resulted in conditions that do not correctly represent the situation in a clinic. Here, we generated progressive FSHD-like pathology in ACTA1-MCM/FLExDUX4 mice and then treated the animals with vivoPMO-PACS4, an antisense compound that efficiently downregulates *DUX4*. To best mimic the translation of this treatment in clinical settings, the systemic antisense oligonucleotide administration was delayed to 3 weeks after the *DUX4* activation so that the pathology was established at the time of the treatment. The chronic administration of vivoPMO-PACS4 for 8 weeks downregulated the *DUX4* expression by 60%. Consequently, the treated mice showed an increase by 18% in body-wide muscle mass and 32% in muscle strength, and a reduction in both myofiber central nucleation and muscle fibrosis by up to 29% and 37%, respectively. Our results in a more suitable model of FSHD pathology confirm the efficacy of vivoPMO-PACS4 administration, and highlight the significant benefit provided by the long-term treatment of the disease.

## 1. Introduction

Facioscapulohumeral muscular dystrophy (FSHD) is a rare hereditary autosomal dominant disease with an estimated prevalence of 5 to 13 per 100,000 [1,2]. As the name suggests, FSHD affects muscles in the face, shoulder girdle and upper arms, and often extends to the trunk and lower limbs as the disease progresses, causing about 20% of patients to become wheelchair-bound [3]. Unlike most muscular dystrophies, the muscle weakness in FSHD is clinically asymmetric [4]. The disease is also gender-specific, with a higher proportion of females than males being asymptomatic or less affected [4]. Although the life expectancy is normal, over 90% of FSHD patients experience chronic fatigue [5]. More than one third of patients have reported respiratory dysfunction, cardiac arrythmias and/or sensorineural hearing loss [6,7,8]. Sleep-related breathing disorders have been additionally identified in at least 40% of patients [9]. In two independent studies involving 80–100 FSHD patients with different disease severities, 14% of the participants required non-invasive ventilation, with the risks of pulmonary insufficiency increasing with spine deformity, severity level and wheelchair dependency [6,7]. However, it remains under debate whether the respiratory impairment in FSHD is caused by weakness of the expiratory muscles and the proximal lower extremities [10] or by weakness of both the expiratory and inspiratory muscles, and in particular the diaphragm [11].

The current treatment options for FSHD are supportive. However, therapeutic agents aiming at slowing or halting disease progression remain to be investigated or verified. Since the aberrant expression of the double homeobox 4 (*DUX4*) gene has been suggested as a predominant cause of FSHD pathogenesis [12,13,14], targeting *DUX4* has great potential to provide a cure for the disease. Therefore, we and others have developed several approaches for suppressing *DUX4* expression or its downstream activity [14,15,16,17,18,19,20,21,22,23,24,25,26,27,28,29,30,31,32,33]. Despite FSHD affecting multiple skeletal muscles, only a few studies describe the systemic effects of therapeutic strategies on FSHD-like animal models. We previously showed that a four- weekly systemic administration of vivoPMO-PACS4, an octaguanidine dendrimer-conjugated phosphorodiamidate morpholino oligomer that targets key features in the 3′UTR of *DUX4*, inhibits *DUX4* expression and improves functionality as well as the molecular and histological features of the muscles of treated ACTA-MCM/FLExDUX4 mice [33,34]. Using the same FSHD-like transgenic mouse model, but without inducing *DUX4* expression, Bouwman and colleagues have since reported that the longitudinal delivery of a constrained ethyl gapmer antisense oligonucleotide targeting the open reading frame of *DUX4* is also effective at knocking down the leaky *DUX4* level and alleviating muscle pathology, although with limited improvement in muscle mass and function [21]. Several pharmaceutical companies have also tested their lead antisense compounds in ACTA1-MCM/FLExDUX4 mice. For example, Arrowhead Pharmaceuticals showed that the systemic delivery of their best siRNA candidate from 2 to 10 days after tamoxifen (TMX)-mediated *DUX4* induction improved the body mass, improved the animal’s performance on the rotarod test and reduced muscle fibrosis [35]. Although the results from these studies are promising, the experimental models used prevent these findings from providing real advances to clinical settings. This is because the treatments were designed to specifically address the leaky *DUX4* level, or they were applied shortly after switching on *DUX4* expression, which is less relevant for FSHD patients who suffer from chronic DUX4 pathogenesis.

Hence, in this study we investigated the long-term inhibitory effect of vivoPMO-PACS4 on *DUX4* in the ACTA1-MCM/FLExDUX4 mouse model by starting the treatment after the DUX4-mediated pathology was already established. We injected the mice with TMX to induce *DUX4* expression and repeated the TMX delivery biweekly to maintain an FSHD-like pathology. A systemic antisense treatment with vivoPMO-PACS4 was started 3 weeks after the *DUX4* activation. The chronic administration of the antisense chemistry for over 8 weeks efficiently suppressed the *DUX4* expression at both the mRNA and protein levels. Consequently, we observed a significant increase in the mass of five examined muscle types, an enhancement of muscle function and an improvement in the histopathological features of the tibialis anterior and diaphragm muscles of the treated mice. Our data therefore confirm the benefit provided by systemic treatment with vivoPMO-PACS4, further highlighting the significant impact of long-term treatment with the conjugated PMO chemistry and the likely relevance to FSHD patients. These results support the further development of the antisense strategy as a treatment for FSHD.

## 2. Materials and Methods

### 2.1. Antisense Chemistries

Phosphorodiamidate morpholino oligomers conjugated to cell-penetrating moiety octaguanidine dendrimers (vivoPMOs) were purchased from GeneTools (Philomath, OR, USA). vivoPMO-PACS4 (AGGATCCACAGGGAGGAGGCATTTTAAT) targets both the polyadenylation signal and the cleavage site of *DUX4* mRNA [33]. vivoPMO-SCR (CCTCTTACCTCAGTTACAATTTATA) is the standard control of GeneTools; it targets the *HBB* mutation that causes β thalassemia, and was used here as a negative control. vivoPMOs were dissolved in sterile ddH_2_O and were further diluted to the desired concentrations in sterile 0.9% saline (Sigma, Dorset, UK) immediately prior to their use in mice.

### 2.2. Animals

This study was conducted in accordance with the UK Animals (Scientific Procedures) Act 1986. Ethical and operational permissions were granted by the UK Home Office (Project Licence P36A9994E) and the Animal Welfare Committee of the Royal Holloway University of London. Mice were bred in a minimal disease facility at the Royal Holloway University of London and kept under a standard 12 h light/dark cycle with free access to food and water. Animal welfare was maintained according to the UK Home Office’s Code of Practice for the Housing and Care of Animals Bred, Supplied or Used for Scientific Purposes. FLExDUX4 (JAX 028710) and ACTA1-MCM (JAX 025750) mice were purchased from The Jackson Laboratory (Maine, USA). The FLExDUX4 colony was maintained as homozygous for Gt(ROSA)26Sor^tm1.1(DUX4*)Plj^ while the ACTA1-MCM colony was maintained as hemizygous for Tg(ACTA1-cre/Esr1*)2Kesr on a C57BL/6J background. A tamoxifen (TMX)-inducible bi-transgenic model named ACTA1-MCM/FLExDUX4 was generated by crossing ACTA1-MCM males with FLExDUX4 females. Genotyping was performed using DNA isolated from the murine ear notches, based on The Jackson Laboratory’s established protocols. Due to the sex specific-DUX4 pathology of the ACTA1-MCM/FLExDUX4 model [36], only males were used in the study and littermates were randomized between experimental groups.

### 2.3. Study Design

This study involved two groups of ACTA1-MCM/FLExDUX4 mice and one group of ACTA1-MCM mice, with 6 mice per group. All animals received the first dose of TMX, prepared as described previously [37], at 1.5 mg/kg via an intraperitoneal (IP) injection at 10 weeks old (considered as week 1 of the study). TMX was re-administered every 2 weeks for a total of 6 times. The ACTA1-MCM/FLExDUX4 mice were further IP injected with 10 mg/kg of either vivoPMO-PACS4 (PACS4) or vivoPMO-SCR (SCR) while the ACTA1-MCM mice received volume-matched saline, which was considered as the positive control (CTRL). Either vivoPMOs or saline was delivered one week after the second TMX dose and repeated once/twice per week thereafter for a total of 12 injections. During the study, the animals underwent several behavioral and functional tests. Treadmill exhaustion tests were conducted one week prior to the first TMX injection and on weeks 3, 7 and 11. Grip strength tests were conducted on weeks 8 and 11. Locomotor behavioral tests were conducted every 2 days during week 11 (prior to any injection or functional test). In situ muscle force measurements and subsequent tissue collection were performed on week 12. Bodyweight was recorded prior to each injection. The mice were kept under isoflurane-induced anesthesia (3% in 100% O_2_) during injections and were continuously monitored until they fully recovered.

### 2.4. Behavioral and Functional Tests

The open-field behavioral activity of the mice was examined using locomotor activity monitors as described in [33]. The data were acquired and collected by Amon Lite software (version 1.4) every 10 min in a one-hour session per test and were summed up for each mouse. The data reported here were an average of 3 tests. Both the locomotor activity monitors and the software were obtained from Linton Instrumentation (Diss, UK). Treadmill exhaustion tests were performed on a Treadmill Simplex II system (Columbus Instruments, Columbus, OH, USA) with adjusted 15 °C inclination as described previously [33]. The total running time was recorded and the time to fatigue was displayed as a percentage of the time recorded in the initial test. Forelimb muscle force was assessed using a commercial grip strength monitor (Linton Instrumentation, Norfolk, UK) as described in [38]. Briefly, mice were held by the tail, allowed to grasp a metal mesh attached to a force transducer with their forelimbs, and then were gently pulled until they released the grip. Measurements were performed 5 times per mouse, with 30 s elapsed between each measurement. The maximal force recorded was expressed as mN per gram of the bodyweight recorded at the assessment. Tibialis anterior (TA) muscle strength was assessed by in situ muscle force measurements, as detailed in [33], while mice were under terminal anesthesia induced by a mixture of 10 mg/mL dolethal (Vetoquinol, Towcester, UK) and 15 µg/mL buprenodale (Dechra, Shrewsbury, UK) at 6 times of their bodyweight. Data were recorded and analyzed using Dynamic Muscle Control and Analysis Software (Aurora Scientific, Aurora, Canada). All isometric measurements were obtained at an initial length at which the maximal tension was recorded during the tetanus. The responses to tetanic stimulations at increased pulse frequencies from 10 Hz to 180 Hz were recorded and the maximal force was determined. The specific force was subsequently calculated based on a ratio of the maximal force and the muscle cross-sectional area (CSA), which was mathematically approximated by dividing the muscle mass by the optimum fiber length and the density of mammalian muscle as described in TREAT-NMD SOP DMD_M.2.2.005.

### 2.5. Post-Mortem Tissue Processing

From each mouse, the diaphragm (DIA), gastrocnemius (GAS), quadriceps (QUAD), TA and triceps (TRI) muscles were dissected and weighed after the fat/connective tissue was trimmed off. The weights of the ipsilateral and contralateral type-matched muscles were then averaged, with the exception of the DIA, and normalized to the corresponding final bodyweight (Table S1). The muscles from one side of the hindlimb were frozen immediately in liquid nitrogen for subsequent RNA extraction. The contralateral muscles were embedded in an optimal cutting temperature (OCT) medium (VWR, Lutterworth, UK) and frozen in liquid-nitrogen-cooled isopentane (Sigma, Dorset, UK) for histological analyses. The DIA muscle was longitudinally divided into 2 halves. One half was frozen immediately in liquid nitrogen for RNA work while the other half was prepared as described in [39] for histological analyses. OCT-embedded frozen muscles were cryosectioned on an OTF5000 cryostat (Bright, Huntingdon, UK) at a 10 µm thickness for 10 serial levels through the muscle length, and the transverse sections were collected onto SuperFrost slides (VWR, Lutterworth, UK).

### 2.6. RT-qPCR Quantification for DUX4 and Relevant Genes

The total RNA from liquid-nitrogen-snap-frozen muscles was isolated using RNeasy Fibrous Tissue kit (QIAgen, Manchester, UK) by following the manufacturer’s instructions. The samples were homogenized in the lysis buffer provided with the kit on a TissueLyser II (QIAgen, Manchester, UK) at 25 Hz for 2–4 min. One microgram of RNA was reverse transcribed using a QuantiTect reverse transcription kit (QIAgen, Manchester, UK). Ten nanograms of diluted cDNA in qPCR water (Roche, Burgess Hill, UK) were then amplified using a LightCycler480 SYBR Green Master I kit (Roche, Burgess Hill, UK) according to the manufacturer’s instructions, with each sample prepared in triplicates. qPCR reactions were run on LightCycler480 System, initialized at 95 °C for 5 min and followed by 45 cycles at 95 °C for 15 s, 58–60 °C for 15 s, and 72 °C for 15 s. The relative quantification for *DUX4* and DUX4-related genes was performed against the corresponding housekeeping gene, *Gapdh*, using Pfaffl’s method as described in [40]. Data are shown as fold-changes compared to the SCR values obtained in the same way. The primers were purchased from Integrated DNA Technologies (Belgium) and are detailed in Table 1.

### 2.7. Immunohistochemistry

For DUX4 and laminin co-immunostaining, frozen muscle sections were fixed in 4% (*w*/*v*) paraformaldehyde for 15 min, permeabilized in 0.3% (*v*/*v*) Triton X-100 with 1× PBS for 10 min and blocked in a buffer containing 2% (*w*/*v*) BSA, 5% (*v*/*v*) goat serum, 0.1% (*v*/*v*) Triton X-100 and 1× PBS for 30 min. An incubation with rabbit anti-DUX4 [E5.5] (1:100, Abcam, UK) and rat anti-laminin (1:1000) antibodies was performed overnight at 4 °C, and then an incubation with goat anti-rabbit AlexaFluor568 and goat anti-rat AlexaFluor488 antibodies (1:400, Life Technologies, Paisley, UK) was performed for 1 h at room temperature. Nuclei were stained with 1 µg/mL DAPI in 1× PBS for 15 min. For collagen immunostaining, the muscle sections were fixed in ice-cold acetone for 10 min and blocked in 1% (*w*/*v*) BSA, 1% (*v*/*v*) goat serum, 0.1% (*v*/*v*) Triton X-100 and 1× PBS for 1 h. A subsequent incubation with rabbit anti-collagen VI antibodies (1:300, Abcam, Cambridge, UK) was carried out overnight at 4 °C, followed by a 1 h incubation with goat anti-rabbit AlexaFluor488 antibodies (1:1000, Life Technologies, Paisley, UK). The slides were mounted in Mowiol 4-88. Ten random images from each muscle section were captured at a magnification of ×200 for DUX4-positive nuclei quantification. For other analyses, multiple images from the largest mid-belly muscle section were captured at a magnification of x100 and were automatically stitched together by ZEN Imaging software (Zeiss, Cambridge, UK), generating an image of the whole transverse muscle section. Images were acquired on an Axio Observer D1 fluorescence microscope by an AxioCam MR3 (Zeiss, Cambridge, UK). For hematoxylin and eosin (H&E) analysis, frozen muscle sections were fixed in 100% (*v*/*v*) ice-cold methanol for 10 min and then submerged in hematoxylin (Vector Laboratories, Peterborough, UK) and eosin solutions. Sections were dehydrated in a series of 50%, 80%, 90% and 100% (*v*/*v*) ethanol washes, for 1 min/wash, and were then cleared twice in 100% (*v*/*v*) xylene for 5 min/wash. The slides were mounted in DPX Mountant. Images were acquired using an Eclipse Ni-E upright microscope and compatible software (Nikon Instruments Inc., New York, NY, USA). The reagents were purchased from Sigma (Dorset, UK) unless stated otherwise.

### 2.8. Histological Analyses

The number of myonuclei that were positive for DUX4 was manually counted using Fiji/MuscleJ software (version 1.53c, National Institutes of Health, Bethesda, MD, USA) and expressed as the total DUX4-positive nuclei per mm^2^ of muscle section. The myofiber perimeter was identified by laminin staining. The subsequent quantifications of the total fiber number, the number of centrally nucleated fibers (CNFs), the area and the minimal Feret’s diameter of individual myofibers were automatically scored by Fiji/MuscleJ software as described in [41]. Automatic analyses of the frequency distribution and the coefficient of variance of the minimal Feret’s diameter were carried out using GraphPad Prism8 software (San Diego, CA, USA). The CSA of the entire muscle sections or the area positive with collagen VI was semi-automatically measured by Fiji/MuscleJ. The fibrotic area was expressed as the percentage of the total area of the muscle CSA.

### 2.9. Statistical Analysis

Data were analyzed using the GraphPad Prism8 software (San Diego, CA, USA) and are shown as the means ± SEM. Error bars represent the SEM; “*n*” refers to the number of mice per group. All data passed the normality Shapiro–Wilk test, which is the most powerful test among four common normality tests, especially for small sample sizes (3 ≤ *n* ≤ 5000) [42]. Comparisons of statistical significance were further assessed by one-way or two-way ANOVA followed by Tukey’s post hoc test, as detailed in figure legends. All functional tests and histological analyses were performed in a blinded manner.

## 3. Results

### 3.1. Systemic Treatment with vivoPMO-PACS4 Increases Muscle Mass of ACTA1-MCM/FLExDUX4 Mice

**Figure 1:**
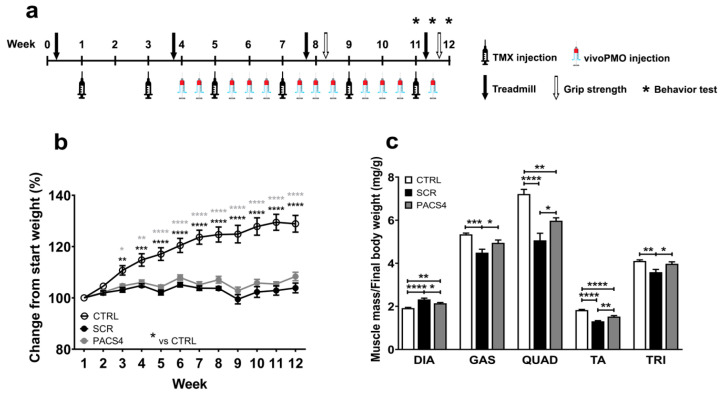
vivoPMO-PACS4 treatment improved body-wide muscle weight. (**a**) Diagram of the experiment, including timepoints of injections of tamoxifen (TMX) to induce *DUX4* expression and vivoPMOs, and functional tests. All mice received 6 × 1.5 mg/kg/injections of TMX via intraperitoneal (IP) delivery. ACTA1-MCM/FLExDUX4 mice were further IP injected with 12 × 10 mg/kg/injections of either vivoPMO-PACS4 (PACS4, *n* = 6) or vivoPMO-SCR (SCR, *n* = 6), considered as a negative control. ACTA1-MCM mice receiving volume-matched saline were considered as a positive control (CTRL, *n* = 6). (**b**) Weekly bodyweight is shown as percentage of the initial weight recorded on the day of the first TMX injection. (**c**) Animals were sacrificed after 8 weeks of vivoPMO treatment. Muscle mass of the diaphragm (DIA), gastrocnemius (GAS), quadriceps (QUAD), tibialis anterior (TA) and triceps (TRI) was normalized to the corresponding final bodyweight. Data are shown as means ± SEM; *n* = 6. Statistical analysis was by (**b**) two-way or (**c**) one-way ANOVA followed by Tukey’s multiple comparisons test; * *p* < 0.05, ** *p* < 0.01, *** *p* < 0.001, **** *p* < 0.0001. In (**b**), black asterisks indicate significance between the SCR and CTRL groups; grey asterisks indicate significance between the PACS4 and CTRL groups.

To best mimic the translation of antisense therapy in clinical settings, in this study we investigated the inhibitory effect of vivoPMO-PACS4 on *DUX4* in ACTA1-MCM/FLExDUX4 mice when the disease pathology was established. Two groups of ACTA1-MCM/FLExDUX4 mice and one group of ACTA1-MCM healthy control mice (six mice/group) received the first tamoxifen (TMX) dose at 1.5 mg/kg via intraperitoneal (IP) injection at 10 weeks of age, and then every two weeks afterwards. Three weeks after the first injection of TMX, the ACTA1-MCM/FLExDUX4 mice were injected (IP) with 10 mg/kg of either vivoPMO-PACS4 (PACS4) or vivoPMO-SCR (SCR), considered as a negative control, while the ACTA1-MCM mice—the positive control (CTRL)—received volume-matched saline. The dosing with vivoPMOs or saline was repeated either once or twice per week, depending on the week and the TMX administration, for a total of 12 injections. Details of the experimental design are shown in Figure 1a. The bodyweight recorded during the study and normalized to the initial weight indicated the successful induction and maintenance of detrimental *DUX4* expression and the related pathology. CTRL mice gained weight gradually up to 130% by the end of the 12-week experiment, whereas the weight gain in the ACTA1-MCM/FLExDUX4 mice fluctuated between 99 and 105% in the SCR group, and between 100 and 108% in the PACS4 group (Figure 1b). Although the PACS4 treatment appeared to have a marginal effect on the bodyweight, we observed a significant improvement in the mass of the five examined skeletal muscles, including the diaphragm (DIA), gastrocnemius (GAS), quadriceps (QUAD), tibialis anterior (TA) and triceps (TRI) (Figure 1c). The muscle mass of the hindlimbs and forelimbs of PACS4-treated mice was significantly increased by up to 18% of the SCR values, achieving the same levels as the CTRL group for the GAS (*p* = 0.7027) and TRI (*p* > 0.9999). Interestingly, the DIA mass of SCR mice was 21% heavier than that of CTRL mice (*p* < 0.0001), and treatment with PACS4 reduced its weight by 8% (*p* = 0.0351 compared to SCR). Hence, systemic treatment with vivoPMO-PACS4 provided a significant benefit to body-wide muscle mass.

### 3.2. Long-Term vivoPMO-PACS4 Treatment Greatly Improves Muscle Function and Animal’s Behavior

**Figure 2:**
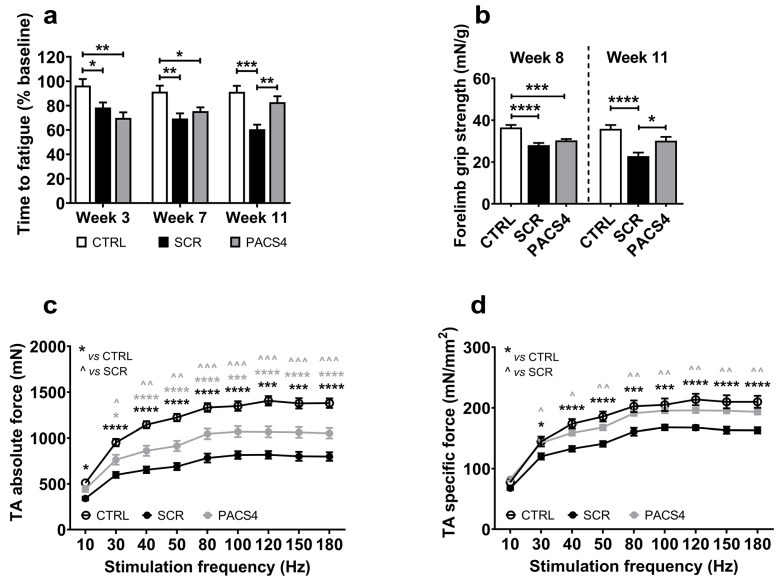
Significant enhancement in muscle function following vivoPMO-PACS4 treatment. (**a**) Treadmill exhaustion tests were conducted 1 week before the first TMX injection, and at weeks 3, 7 and 11. The time to fatigue is the total running time in each test, expressed as percentage of the initial time. (**b**) Forelimb grip strength tests were carried out on weeks 8 and 11. Data are shown as the force corrected to the bodyweight recorded at the assessment. (**c**) In situ absolute force of TA muscles was measured while animals were under terminal anesthesia on week 12. (**d**) Specific muscle force is displayed as a ratio of the absolute force and the muscle cross-sectional area (CSA), mathematically approximated by dividing the muscle mass by the optimum fiber length and the density of mammalian muscle. Data are shown as means ± SEM; *n* = 6. Statistical analysis was by (**a**,**b**) one-way or (**c**,**d**) two-way ANOVA followed by Tukey’s multiple comparisons test; */^ *p* < 0.05, **/^^ *p* < 0.01, ***/^^^ *p* < 0.001, **** *p* < 0.0001. In (**c**,**d**), black asterisks indicate significance between the SCR and CTRL groups; grey asterisks or carets indicate significance between the PACS4 and CTRL or the PACS4 and SCR groups, respectively.

We evaluated the impact of chronic *DUX4* expression and the long-term effect of PACS4 treatment on whole-body muscle function over a 12-week study using the treadmill exhaustion test. Mice were allowed to run on a treadmill until they were unable to move from the stopper for 10 s. The time to fatigue shown in Figure 2a is the total running time at each timepoint calculated as a percentage of the time recorded at the beginning of the experiment. Following two doses of TMX (week 3), the time to reach exhaustion in the ACTA1-MCM/FLExDUX4 mice decreased to 78% (SCR) and 70% (PACS4) of the initial levels, while no reduction was observed in the CTRL mice, confirming the negative impact of the inducible *DUX4* expression in both ACTA1-MCM/FLExDUX4 groups. With continual TMX delivery, the level of resistance to fatigue in the SCR mice decreased further to 69% and 60% on weeks 7 and 11, respectively. However, at the same timepoints, the mice receiving PACS4 displayed an increase in resistance to fatigue, at 75% and 83%, respectively. This means that the fatigue in the PACS4 mice decreased by 23% (*p* = 0.0030) compared to SCR mice, and that the PACS4-treated mice were as healthy as the CTRL mice (*p* = 0.3686) by the last vivoPMO delivery. An additional assessment of the strength of the forelimb muscles using the grip strength test (Figure 2b) indicated that the forelimbs of the PACS4 mice were as strong as those of the SCR mice (30.3 ± 0.7 mN/g vs. 28.1 ± 1.0 mN/g, *p* = 0.6725) on week 8. While the forelimb muscle force in the SCR mice dropped by a further 19% on week 11, the value in the PACS4 mice remained unchanged (30.2 ± 1.9 mN/g) and was similar to the CTRL value (35.9 ± 1.8 mN/g, *p* = 0.0770). Hence, the vivoPMO-PACS4 treatment prevented a 32% loss of forelimb strength compared to the SCR level (*p* = 0.0180). Further in situ TA force measurements demonstrated obvious muscle weakness in the ACTA1-MCM/FLExDUX4 mice receiving vivoPMO-SCR when compared to the CTRL group (Figure 2c,d). Treatment with PACS4 significantly improved the maximal tetanic force, from 798.1 ± 46.8 mN measured in SCR mice to 1051.8 ± 57.1 mN (*p* = 0.0006) at the highest stimulation frequency of 180 Hz. Notably, mice receiving PACS4 exhibited a specific maximal force comparable to CTRL mice, with 193.4 ± 4.3 mN/mm^2^ and 209.9 ± 9.9 mN/mm^2^ at 180 Hz, respectively (*p* = 0.1566), which corresponded to a 19% increase compared to the strength of the SCR group’s muscles (*p* = 0.0025).

The efficacy of the vivoPMO treatment was also assessed using open-field activity cage monitors a week before the end of the study. The locomotor behavior of the mice injected with vivoPMO-SCR significantly decreased in 19 of the 22 parameters examined, compared to CTRL mice (Table 2). In contrast, the PACS4-treated animals exhibited a similar behavior to that of the CTRL group in all measurements, and a robust improvement in six parameters as compared to the SCR mice. Notably, the total activity, total rearing counts and rearing time were all increased from 361 ± 51 to 565 ± 52 beam breaks (*p* = 0.0085), 86 ± 20 to 247 ± 29 beam breaks (*p* = 0.0050) and 148 ± 31 to 449 ± 53 s (*p* = 0.0392), respectively. Taken together, these data suggest that chronic vivoPMO-PACS4 treatment has extensive benefits on muscle function and behavior, efficiently stabilizing the disease progression in the treated mice.

### 3.3. vivoPMO-PACS4 Robustly Downregulates Expression of DUX4 and Murine DUX4-Related Targets

**Figure 3:**
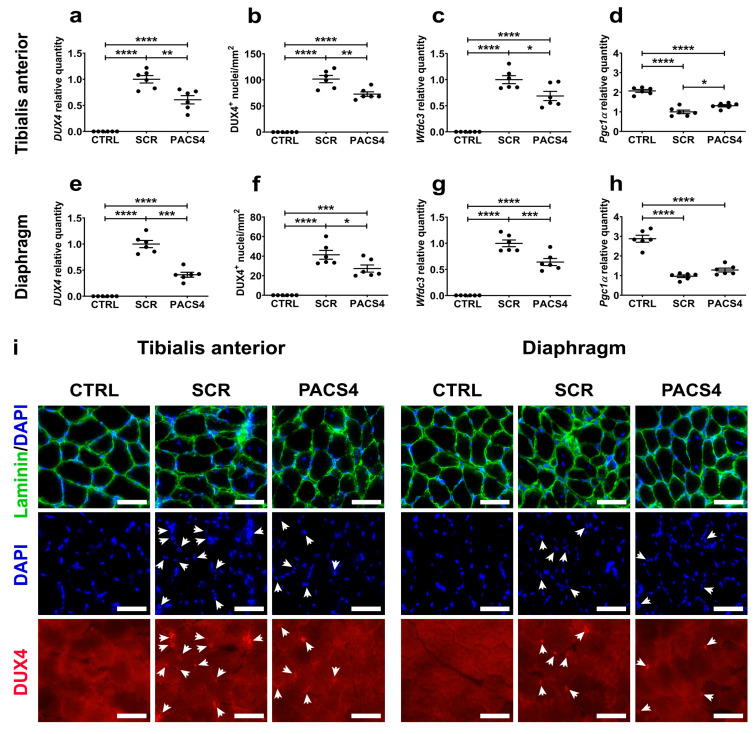
vivoPMO-PACS4 treatment downregulated the expression of *DUX4* and murine DUX4-related genes. Following chronic TMX-induced *DUX4* expression and an 8-week treatment with vivoPMOs, mRNA levels of *DUX4* and DUX4-related genes, and DUX4 protein levels in (**a**–**d**) the tibialis anterior and (**e**–**h**) the diaphragm muscles were evaluated. mRNA expression of (**a**,**e**) *DUX4* and (**c**,**d**,**g**,**h**) DUX4-related genes was quantified by qRT-PCR as relative to the corresponding *Gapdh* level and expressed as a fold-change of the SCR value obtained by the same way. (**b**,**f**) Muscle sections were immunostained with DUX4 and laminin, and nuclei were stained with DAPI. The number of myonuclei positive with DUX4 was manually counted and expressed as the total DUX4+ nuclei per mm^2^ of the transverse muscle section. Data are shown as means ± SEM; *n* = 6. Statistical comparison was by one-way ANOVA followed by Tukey’s multiple comparisons test; * *p* < 0.05, ** *p* < 0.01, *** *p* < 0.001, **** *p* < 0.0001. (**i**) Representative images are shown at a magnification of ×200; scale bars = 50 µm; white arrows indicate DUX4+ nuclei.

Among the five muscle types examined here, the TA and DIA muscles of ACTA-MCM/FLExDUX4 mice have been shown to be the most affected by *DUX4* expression via systemic TMX-mediated induction [33,34,36]. Hence, our investigation on the effect of vivoPMO-PACS4 against DUX4-mediated pathology at the molecular and histological levels focused on these two muscles. qRT-PCR quantification demonstrated that PACS4 treatment efficiently suppressed the *DUX4* mRNA expression by 40% in the TA (*p* = 0.001) and by 60% in the DIA (*p* < 0.0001), compared to the corresponding SCR levels (Figure 3a,e). This led to a reduction in the number of DUX4-positive nuclei per mm^2^ of muscle section from 101.5 ± 6.9 (TA) and 41.4 ± 4.5 (DIA) in the SCR group to 72.8 ± 4.3 (TA, *p* = 0.0017) and 27.3 ± 3.7 (DIA, *p* = 0.0249) in the PACS4 group (Figure 3b,f,i). As a consequence, the mRNA expression of a murine DUX4-related target, *Wfdc3* [36], in PACS4 muscle was downregulated by 32% in the TA (*p* = 0.0131) and by 35% in the DIA (*p* = 0.0008), compared to the SCR group’s levels (Figure 3c,g). Moreover, in agreement with previous findings suggesting that DUX4 interferes with mitochondrial biogenesis via suppressing the expression of a key mediator, *Pgc1α* [40], we detected a significantly lowered *Pgc1α* level in the SCR group by 52% (TA, *p* < 0.0001) and by 69% (DIA, *p* < 0.0001), compared to the CTRL values. Treatment with PACS4 significantly improved the *Pgc1α* expression by 31% in the TA muscle (*p* = 0.0251), but had a minimal effect on the expression in the DIA (*p* = 0.1958) compared to the SCR treatment (Figure 3d,h). These results confirm the efficacy of the systemic delivery of vivoPMO-PACS4 on *DUX4* at both the mRNA and protein levels, as well as on the DUX4 downstream target genes.

### 3.4. Long-Term vivoPMO-PACS4 Treatment Greatly Improves Muscle Histopathology

**Figure 4:**
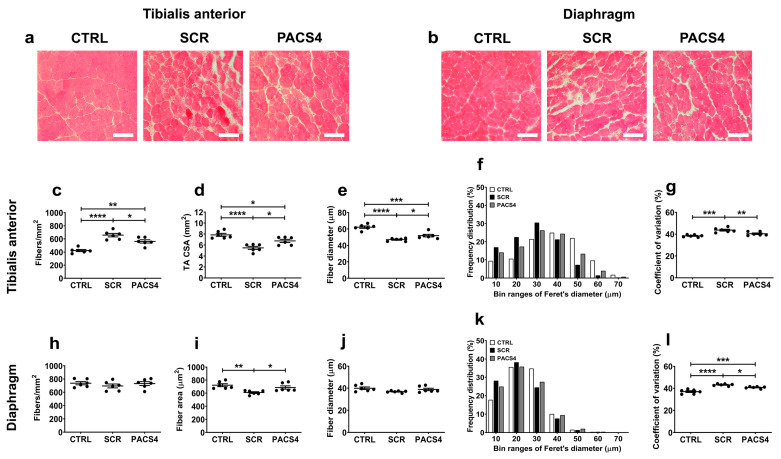
Improved muscle histopathology following longitudinal vivoPMO-PACS4 treatment. Histopathological changes in (**a**) the tibialis anterior (TA) and (**b**) the diaphragm (DIA) muscles were initially assessed by hematoxylin and eosin analysis. Representative images are shown at a magnification of ×100, scale bars = 100 µm. Both muscles were further stained for laminin to identify the myofiber sarcolemma. The total number and size of (**c**–**g**) TA and (**h**–**l**) DIA myofibers were automatically scored by Fiji/MuscleJ software (version 1.53c, National Institutes of Health, Bethesda, MD, USA). (**c**,**h**) The fiber number per mm^2^ of the muscle CSA, (**d**) the area of whole transverse TA muscle or (**i**) area of DIA fibers, (**e**,**j**) the mean of minimal Feret’s diameter of fibers, (**f**,**k**) a histogram of the frequency distribution of the fiber diameter and (**g**,**l**) the coefficient of variance of the fiber diameter are shown for the TA and DIA, respectively. Data are shown as means ± SEM; *n* = 6. Statistical comparison was by one-way ANOVA followed by Tukey’s multiple comparisons test; * *p* < 0.05, ** *p* < 0.01, *** *p* < 0.001, **** *p* < 0.0001.

The impact of chronic *DUX4* expression on the histology of the TA and DIA muscles was first assessed through hematoxylin and eosin (H&E) staining (Figure 4a,b). The SCR group’s muscles displayed substantial deterioration in the muscle architecture and quality, with increased fiber size variation, central nucleation, fibrosis, and necrosis/inflammation compared to the CTRL group’s muscles. The hallmarks of muscle fibrosis and necrosis/inflammation were particularly more abundant in the DIA muscle than in the TA muscle, which may account for the pseudo-hypertrophy in the mass of the DIA contrary to the atrophy in the TA mass, as presented in Figure 1c. In contrast, the PACS4-treated tissues exhibited a histology similar to that of the CTRL muscles, which was consistent with the improvement in the muscle mass towards a value comparable to the CTRL group’s values. The immunohistochemical detection of laminin in these muscle sections provided additional information about the pathology process. The TA muscle appeared to have undergone a significant process of muscle deterioration and regeneration (Figure 4c–g). We observed a high increase in the number of myofibers (*p* < 0.0001), but a significant reduction in both the cross-sectional area (CSA) and the myofiber diameter of the SCR-treated muscle (*p* < 0.0001), as compared to the CTRL group. The PACS4 treatment significantly reduced the myofiber number by 25% (*p* = 0.0248) while increasing the CSA by 23% (*p* = 0.0158) and the fiber diameter by 10% (*p* = 0.0410) compared to the SCR values (Figure 4c–e). Accordingly, this led to an 8% reduction in the coefficient of variation for the fiber diameter in the SCR muscle (*p* = 0.0097), completely normalizing the fiber diameter to the level of the CTRL muscles (*p* = 0.1422) (Figure 4f,g).

Surprisingly, in contrast to the remarkable changes observed through H&E staining, we detected only a minor decrease in both the number and the diameter of the DIA myofibers of the SCR group, compared to the CTRL and PACS4 values (Figure 4h,j). However, the area of myofibers in the SCR group’s DIA was significantly smaller than that of the CTRL group’s myofibers by 16% (*p* = 0.0046, Figure 4i), while the coefficient of variation increased from 36.9 ± 0.8% to 43.1 ± 0.4% (*p* < 0.0001, Figure 4k,l). The PACS4 treatment successfully restored the myofiber area to a normal level (*p* = 0.4684) and increased the coefficient of variation to 40.7 ± 0.4% compared to the SCR group (*p* = 0.0232). These results clearly demonstrate that the PACS4 treatment was effective in counteracting DUX4 pathology in both the TA and DIA muscles of treated ACTA-MCM/FLExDUX4 mice.

### 3.5. vivoPMO-PACS4 Treatment Significantly Reduces Muscle Turnover and Muscle Fibrosis

**Figure 5:**
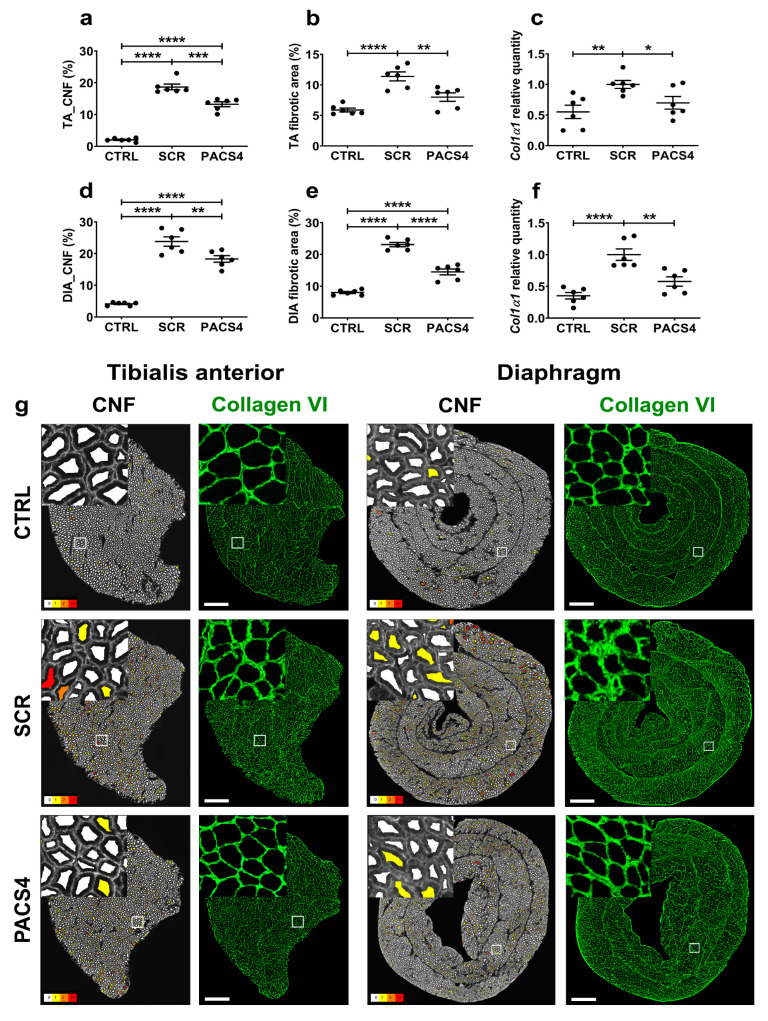
vivoPMO-PACS4 treatment reduces muscle deterioration and muscle fibrosis. The tibialis anterior (TA) and diaphragm (DIA) muscle sections were stained for laminin or collagen VI; DAPI was used for nuclear staining. The total number of centrally nucleated myofibers (CNFs) of (**a**) TA and (**d**) DIA muscles was scored automatically and is expressed as a percentage of the total myofiber number within the same transverse muscle section. Fibrotic area in (**b**) TA and (**e**) DIA muscles was semi-automatically evaluated and expressed as percentage of the area positive for collagen VI of the muscle CSA. mRNA expression of *Col1α1* indicative for fibrotic response in (**c**) TA and (**f**) DIA muscles was quantified by qRT-PCR as relative to *Gapdh* and shown as a fold-change of SCR values obtained by the same way. Data are shown as means ± SEM; *n* = 6. Statistical comparison was by one-way ANOVA followed by Tukey’s multiple comparisons test; * *p* < 0.05, ** *p* < 0.01, *** *p* < 0.001, **** *p* < 0.0001. (**g**) Representative images of the entire muscle cross-sections analyzed by Fiji/MuscleJ for CNFs, with fibers having 0, 1, 2 and 3+ CNFs color-coded as white, yellow, orange and red, respectively. Images of transverse muscle sections immunostained with collagen VI are shown at a magnification of ×100, scale bar = 500 µm. Corresponding enlarged images at higher magnification are shown in the subsets.

Since muscle regeneration in FSHD is correlated with DUX4 pathological severity [43], we further quantified the number of centrally nucleated fibers (CNFs) in both the TA and DIA muscles (Figure 5a,d,g). While the CTRL muscles displayed only 2–4% CNFs in both the TA and the DIA, we detected a substantial increase (*p* < 0.0001) in CNFs for the SCR group’s TA (18.7 ± 0.9%) and DIA (23.8 ± 1.5%). The treatment with PACS4, although it was started 3 weeks after *DUX4* activation, prevented the muscle turnover. Indeed, the PACS4-treated muscles displayed a significant reduction in CNFs by up to 29% of the SCR group (13.2 ± 0.7% CNFs in TA and 18.3 ± 1.0% CNFs in DIA, *p* = 0.0001).

In addition to muscle deterioration, excessive muscle fibrosis negatively affects muscle function and is an important hallmark of FSHD [44]. We therefore additionally examined the level of fibrosis in TA and DIA muscles using immunostaining for a commonly used marker of fibrosis, collagen VI [45,46] (Figure 5b,e,g). Collagen VI quantification revealed that the *DUX4* expression led to an increase in the fibrotic area, from 5.9 ± 0.3% (CTRL) to 11.9 ± 0.7% (SCR, *p* < 0.0001) in the TA and from 8.0 ± 0.3% (CTRL) to 23.1 ± 0.7% (SCR, *p* < 0.0001) in the DIA. The PACS4-treated TA and DIA muscles displayed 8.0 ± 0.7% and 14.5 ± 0.9% of their area covered by collagen VI, respectively, which was a remarkable decrease by up to 37% compared to the fibrosis seen in the SCR muscles (*p* < 0.0001). In agreement with histological analyses, the mRNA level of *Col1α1*, another indicator of a fibrotic response, was highly elevated in both the SCR-treated TA (*p* = 0.0053) and DIA (*p* < 0.0001), compared to the CTRL values. The PACS4 treatment efficiently reduced the *Col1α1* expression to the CTRL level by 30% (*p* = 0.0332) and 42% (*p* = 0.0026) as compared to the levels seen in the SCR-treated TA and DIA, respectively (Figure 5c,f).

The consistency of our data describing improvements in the muscle mass, muscle function, molecular features and histopathology demonstrates that vivoPMO-PACS4 has significant efficacy in inhibiting DUX4 toxicity and stabilizing the disease progression in a chronic ACTA1-MCM/FLExDUX4 model with established pathology.

## 4. Discussion

Despite being the third most common muscular dystrophy, no existing disease-modifying treatment is available for FSHD. With remarkable progress over the last two decades in the understanding of FSHD pathogenesis and the contribution of abhorrent *DUX4* expression, numerous *DUX4*-silencing strategies have been extensively developed [14,15,16,17,18,19,20,21,22,23,24,25,26,27,28,29,30,31,32,33]. Surprisingly, although clinical application requires systemic *DUX4* inhibition, only few research groups (including ours) have investigated the potential systemic approaches in FSHD disease models [21,30,33,34]. In this work, we verified the inhibitory effect of vivoPMO-PACS4 in the transgenic ACTA1-MCM/FLExDUX4 mouse model of FSHD. We demonstrated for the first time that delivering vivoPMO-PACS4 therapy when the pathology is established, which is more relevant for clinical translation, remains effective in downregulating chronic *DUX4* expression at both the mRNA and protein levels in the two most affected skeletal muscles. Furthermore, long-term systemic antisense treatment substantially ameliorates the mass of five muscle types and multiple muscle functions by efficiently stabilizing the disease progression.

ACTA1-MCM/FLExDUX4 is the most used model [21,25,33,34,47] among three transgenic FSHD-like mouse models recently developed [36,48,49]. Despite patients usually being diagnosed with progressive DUX4 pathology, translational research has so far been trialed either in uninduced-*DUX4* models [16,21,32], shortly after *DUX4* induction [30,33,34] or even before *DUX4’s* activation [25]. Several groups have suggested that, without TMX-mediated activation, the leaky mosaic levels of *DUX4* accumulating over the animal’s lifetime are sufficient enough to cause muscle damage and recapitulate FSHD pathogenesis, potentially more than when *DUX4* is forcedly expressed [21,47]. These chronic models indeed provide an advantage for long-term translational studies, offering consistency and reproducibility among research groups. However, mature stages are required in these models (≥3 months old, equivalent to human age ≥20 years) [47] that are unsuitable for investigating the pathological mechanisms involved in infantile FSHD [50]. Recently, the effect of the systemic delivery of gapmer antisense chemistry was evaluated in non-*DUX4*-induced ACTA1-MCM/FLExDUX4 mice for either 4 or 10 weeks [21]. Although substantial downregulation in the expression of the leaky *DUX4* and improvement in some histological features were observed, no changes in muscle mass or muscle function were detected. The authors suggested that this lack of benefit was possibly due to the starting of antisense administration after muscle damage had already occurred, or because females were used, in which the disease is considered more severe. However, using the same mouse model but with continual TMX-mediated *DUX4* expression, we [33,34] and others [35] achieved both *DUX4* knockdown and amelioration in the muscle/body mass, muscle function and/or muscle histology. In previous studies, we used 16-week-old male mice and inducible *DUX4* expression that has been proven to cause more severe pathology than the leaky level, regardless of the animal’s sex [36]. As presented here, even under chronically inducible conditions, our vivoPMO-PACS4 sufficiently counteracted the DUX4 pathogenesis at both the molecular and histological levels, and greatly ameliorated the muscle atrophy and muscle strength. Therefore, our work suggests that inducing *DUX4* expression in ACTA1-MCM/FLExDUX4 mice may make the model more suitable for preclinical tests of antisense therapeutics. However, the TMX dosage for inducing *DUX4* expression requires careful refinement to avoid a burst-like effect that is highly lethal and/or lasts for a short time due to the effective muscle regeneration in rodents, which does not mimic the chronic situation found in FSHD patients [36]. Hence, the dose regimen based on a low chronic TMX dosage presented in this study is more suitable for allowing researchers to perform experiments at an early stage and possibly for long time periods, for example 12 weeks as we used here. Intriguingly, the low continual TMX dosage of 6 × 1.5 mg/kg/biweekly used in this study seemed to cause more muscle damage than the dosage of 2 × 2.5 mg/kg/biweekly previously used in our 4-week study [33,34]. Although we did not observe major drops in the body weight or fatigue resistance as in the short-term experiments, the histopathological changes in the muscles of vivoPMO-SCR-treated mice, particularly in the DIA, appeared to be more severe in this longitudinal study. This may account for the pseudo-hypertrophy seen in the DIA compared to the atrophy seen in other hind/forelimb muscles. Together with the mRNA quantification of *Pgc1α* expression, which showed a substantial reduction in the DIA compared to the TA, the greater severity in the former muscle may provide an explanation for the respiratory dysfunction in FSHD patients [6], although this requires further investigation. Interestingly, the number of DUX4-positive nuclei in DIA tissue in this study was 7-fold less than the amount observed previously [34]. We speculate that the low *DUX4* level in this chronic model did not cause a burst-like effect, but instead transiently triggered a more severe cumulative pathology. This is consistent with an analysis of FSHD muscle biopsies, where DUX4 protein could be found in some samples, but was never detected in those of severely affected patients [51], emphasizing the impact of long-term vivoPMO-PACS4 treatment in a model that more closely represents the human condition.

RNA-silencing therapies have emerged over the last decade as a hot spot for new drug development. A total of fifteen FDA-approved drugs are on the market and more than 100 are in clinical trials (https://clinicaltrials.gov/; accessed on 20 May 2022). Among these, four drugs (EXONDYS 51^®^, VYONDYS 53^®^, VILTEPSO^®^ and SPINRAZA^®^) receiving FDA conditional approval for neuromuscular dystrophies are all based on PMO chemistry. Despite a high biosafety profile and promising therapeutic efficacy [52], the limited cellular uptake due to the neutral charge of the backbone is a major challenge for clinical applications of PMO, particularly for FSHD. Nevertheless, as demonstrated here, vivoPMO chemistry with a PMO backbone conjugated with a cell-penetrating octaguanidine dendrimer effectively penetrates the skeletal muscles, though the development of safer systems to improve tissue penetration will be necessary for the translation of antisense therapeutics into the clinic. Alternatively, the PMO-PACS4 sequence could be conjugated with other cell-penetrating peptides, such as Sarepta Therapeutics’ PPMO chemistry, which has displayed positive results in a phase 2 clinical trial for Duchenne muscular dystrophy (company’s report: https://investorrelations.sarepta.com/news-releases/news-release-details/sarepta-therapeutics-reports-positive-clinical-results-phase-2; accessed on 20 May 2022), or with monoclonal antibodies targeting the transferrin receptor to enable muscle delivery, such as those being trialed in FSHD animal models by Avidity Biosciences or Dyne Therapeutics [35]. Moreover, a comparison of the antisense efficiency between different systemic delivery routes should be conducted, for example between the intraperitoneal route used here and the intravenous or subcutaneous delivery that has been used in clinical settings, to obtain the best therapeutic benefit with the highest clinical relevance.

## 5. Conclusions

In summary, the present study provides substantial evidence that long-term systemic vivoPMO-PACS4 treatment in a mouse model of FSHD, with established disease, efficiently stabilizes the pathology progression through suppressing the expression of *DUX4* and its downstream targets. The observed improvements in body-wide muscle wasting, muscle weakness and muscle histopathology are particularly important, as this data was generated in a mouse model that closely represents the human condition. Overall, our data support further development of this antisense strategy for FSHD therapy.

## Figures and Tables

**Table 1:** Details of qRT-PCR primers.

Target Gene	Accession Number	Primer Sequence (5′-3′)	Amplicon Size (bp)
*DUX4*	Gene ID: 100288687	Forward: CTCTGTGCCCTTGTTCTTC Reverse: TCCAGGAGATGTAACTCTAATCCA	98
*Gapdh*	NM_008084	Forward: TCCATGACAACTTTGGCATTG Reverse: TCACGCCACAGCTTTCCA	103
*Col1α1*	NM_007742	Forward: GAAACTTTGCTTCCCAGATGTC Reverse: AGACCACGAGGACCAGAA	94
*Pgc1α*	NM_008904	Forward: GGCACCTGAACAGAACGAAC Reverse: CAACAGGCATCAGCAGTGTC	180
*Wfdc3*	NM_027961	Forward: CTTCCATGTCAGGAGCTGTG Reverse: ACCAGGATTCTGGGACATTG	134

**Table 2:** Changes in mouse locomotor behavior after longitudinal vivoPMO treatment.

Open-Field Cage Activity		CTRL	SCR	PACS4	SCR vs. WT	PACS4 vs. WT	PACS4 vs. SCR
Parameter	Description	Mean ± SEM	Mean ± SEM	Mean ± SEM	*p* Value	*p* Value	*p* Value
Total activity	Total beam breaks	840 ± 235	361 ± 51	565 ± 52	0.0208 *	0.1976	0.0085 **
Fast activity	Fast beam breaks	87 ± 30	25 ± 4	62 ± 7	0.0209 *	0.4994	0.1803
Slow activity	Slow beam breaks	753 ± 206	336 ± 53	503 ± 50	0.0303 *	0.2209	0.5601
Total static counts	Total beam breaks, movement lower than mobile threshold	636 ± 160	324 ± 52	441 ± 47	0.0449 *	0.2829	0.5705
Fast static counts	Beam breaks, movement lower than mobile threshold and faster than fast threshold	27 ± 8	12 ± 3	20 ± 4	0.1024	0.6527	0.3918
Slow static count	Beam breaks, movement lower than mobile threshold and slower than fast threshold	609 ± 152	312 ± 50	421 ± 43	0.0438 *	0.2690	0.5849
Total mobile counts	Total beam breaks, movement greater than mobile threshold	204 ± 79	48 ± 6	113 ± 11	0.0169 *	0.2257	0.4024
Fast mobile counts	Beam breaks, movement greater than mobile threshold and faster than fast threshold	60 ± 23	14 ± 2	41 ± 5	0.0187 *	0.4972	0.1662
Slow mobile counts	Beam breaks, movement greater than mobile threshold and slower than fast threshold	144 ± 57	34 ± 4	72 ± 8	0.0191 *	0.1607	0.5547
Total rearing counts	Number of rearing beam breaks	242 ± 61	86 ± 20	247 ± 29	0.0199 *	0.9640	0.0050 **
Fast rearing counts	Number of fast rearing beam breaks	91 ± 19	49 ± 15	129 ± 15	0.1875	0.2508	0.0019 **
Slow rearing counts	Number of slow rearing beam breaks	151 ± 37	37 ± 7	118 ± 14	0.0007 ***	0.4825	0.0091 **
Total center rearing counts	Number of rearing beam breaks occurring away from the cage walls	79 ± 23	19 ± 6	70 ± 17	0.0290 *	0.9171	0.0457 *
Fast center rearing counts	Number of fast rearing beam breaks occurring away from the cage walls	34 ± 12	12 ± 5	47 ± 16	0.4262	0.7782	0.0985
Slow center rearing counts	Number of slow rearing beam breaks occurring away from the cage walls	45 ± 14	7 ± 2	23 ± 5	0.0030 **	0.1321	0.2268
Active time	Time of mobile or static activity (s)	585 ± 143	282 ± 42	435 ± 45	0.0286 *	0.3816	0.3216
Static time	Time of static activity (s)	489 ± 112	252 ± 40	369 ± 39	0.0397 *	0.3984	0.3802
Mobile time	Time of mobile activity (s)	96 ± 34	30 ± 4	66 ± 7	0.0222 *	0.4175	0.2468
Rearing time	Time spent rearing (s)	570 ± 170	148 ± 31	449 ± 53	0.0062 **	0.6193	0.0392 *
Front to back counts	Number of traverses from front to back	50 ± 15	14 ± 2	33 ± 3	0.0054 **	0.2534	0.1697
Inactive time	Time spent in inactivity (s)	2992 ± 153	3290 ± 49	3166 ± 45	0.0382 *	0.3090	0.4704
Distance travelled	Total distance travelled (m)	35 ± 8	17 ± 3	31 ± 3	0.0400 *	0.8487	0.0904

Data were assessed by GraphPad Prism8 (San Diego, CA, USA). Statistical significance was analyzed by one-way ANOVA followed by Tukey’s post hoc test, *n* = 6, * *p* < 0.05, ** *p* < 0.01, *** *p* < 0.001.

## Data Availability

Not applicable.

## Supplementary Materials

The following supporting information can be downloaded at: https://www.mdpi.com/article/10.3390/biomedicines10071623/s1, Table S1: Muscle and body weight.
