# Supplementary material for: Long-Term Systemic Treatment of a Mouse Model Displaying Chronic FSHD-like Pathology with Antisense Therapeutics That Inhibit DUX4 Expression
Source: Biomedicines. 2022 Jul 7;10(7):1623. doi: 10.3390/biomedicines10071623 (PMC9313434; doi:10.3390/biomedicines10071623)
Supplement: Supplementary file 1 [file biomedicines-10-01623-s001.zip › biomedicines-1789322-supplementary.pdf]

Table S1: Muscle and body weight

Group	Initial body weight (g)	Final body weight (g)	DIA weight (mg)	GAS weight (mg)	QUAD weight (mg)	TA weight (mg)	TRI weight (mg)
CTRL	20.8	28.6	52.1	155.6	227.1	52.9	113.0
CTRL	23.7	30.3	59.1	166.7	226.0	56.9	123.9
CTRL	23.0	26.7	49.9	136.4	173.0	45.5	111.1
CTRL	20.9	26.2	51.1	140.9	183.4	47.3	104.0
CTRL	24.1	31.2	64.0	165.1	214.0	60.0	132.0
CTRL	22.1	30.3	57.0	162.1	228.5	54.7	128.5
SCR	26.2	26	60.1	122.4	146.4	34.4	100.1
SCR	25.0	27.8	63.9	112.6	117.0	35.8	85.6
SCR	25.1	25.9	66.1	111.1	109.1	34.9	93.0
SCR	29.6	29.4	62.9	140.5	173.2	38.9	116.4
SCR	27.8	28.8	70.0	119.2	133.6	34.3	102.0
SCR	27.2	29.1	64.0	145.5	169.4	40.5	102.1
PACS4	27.7	28.4	63.0	148.0	172.7	45.7	119.0
PACS4	24.4	27.1	58.3	123.0	150.7	38.6	101.1
PACS4	27.2	29.5	59.0	149.0	179.4	42.6	121.5
PACS4	22.3	23.9	53.5	122.9	148.2	39.7	99.9
PACS4	21.2	24.3	51.5	111.0	135.6	34.2	95.5
PACS4	28.9	30.8	65.9	160.2	195.6	49.4	114.0

DIA = diaphragm, GAS = gastrocnemius, QUAD = quadriceps, TA = tibialis anterior, TRI = triceps
